# Intertwining extracorporeal membrane oxygenation and continuous renal replacement therapy: sense or nonsense?

**DOI:** 10.1186/s13054-015-0860-6

**Published:** 2015-03-25

**Authors:** Rita Jacobs, Patrick M Honore, Herbert D Spapen

**Affiliations:** ICU Department, UZ Brussel, VUB University, 101 Laarbeeklaan, 1090 Jette, Brussels, Belgium

We would like to comment on the excellent review by Chen and colleagues highlighting the safety and efficacy of combining extracorporeal membrane oxygenation (ECMO) and continuous renal replacement therapy (CRRT) for fluid and electrolyte control [1].

As a high-flow system equipped with heparin-coated membranes and circuits, ECMO requires no or only minimal additional anticoagulation to assure circuit patency [2,3]. In contrast, CRRT essentially remains a low-flow system that demands specific anticoagulation to avoid early circuit clotting [2,3]. Thus, creating a two-in-one ECMO/CRRT system will have evident limitations and drawbacks. The introduction of a hemofiltration filter inside the ECMO circuit renders ultrafiltrate volume removal inaccurate and prohibits pressure monitoring in the circuit, thereby reducing the filter lifespan. Embedding a full CRRT device in series with an ECMO circuit may obviate these shortcomings. However, the dramatic difference in flow and pressure will increase shear stress, activate the clotting cascade and release noxious cytokines, which exposes patients to the potential life-threatening effects of hemolysis, disseminated intravascular coagulation and enhanced systemic inflammation [4,5]. Moreover, a hemolysis-induced excessive rise of plasma-free hemoglobin and subsequent hemoglobinuria adversely affects renal tubular function and may induce or exacerbate acute kidney injury [4,5].

For these reasons, we strongly argue against the combined use of ECMO and CRRT within a single circuit. In addition, a separate CRRT device can perfectly run under a proper dedicated anticoagulation therapy (for example, regional citrate). This permits avoidance of ECMO-induced anticoagulant dilution, resulting in less thrombotic events [2].

## Abbreviations

CRRT: Continuous renal replacement therapy
ECMO: Extracorporeal membrane oxygenation
